# Comment on ‘Will Earth's next supercontinent assemble through the closure of the Pacific Ocean?’

**DOI:** 10.1093/nsr/nwac253

**Published:** 2022-11-09

**Authors:** Bernhard Steinberger

**Affiliations:** GFZ German Research Centre for Geosciences, Germany; Centre for Earth Evolution and Dynamics, University of Oslo, Norway

In their paper, Huang *et al.* show that both introversion and extroversion can occur within the same model, if parameters are changed. This by itself is an important result, however, the authors go further to conclude which continents assembled with introversion and which ones with extroversion. This conclusion rests on the assumption that a thicker crust leads to a stronger lithosphere, but this is not necessarily the case. The experimental strength profile shown in their Figure S8 is in fact not used in their modeling; like other numerical models, they had to assume a weakening mechanism in order to obtain a plate-tectonic-like behavior. Accordingly, the authors basically make the assumption that the mantle is weaker than in the experiments, and the crust is not so much (or not at all) weaker, and that hence a thicker crust gives a stronger lithosphere. But why would that be the case? If anything, the typical ‘Christmas tree’ diagrams of lithospheric strength profiles rather show the opposite—that thicker crust gives a weaker overall lithosphere. Besides, there are also other processes affecting lithospheric strength, one being that a hotter lithosphere is thermally weaker, another that a hotter temperature leads to more melting, and the residue in the mantle in this case can again lead to a stronger lithosphere. Given all these contrasting effects, it remains unclear whether the oceanic lithosphere would have been stronger or weaker during earlier times. The authors also make the counterintuitive statement that the thickness of the oceanic lithosphere decreases as the Earth cools with time.

Another assumption made is the *ad hoc* lowering of strength when ‘older’ oceanic lithosphere is next to a continent. It is not clear to what extent results are primarily due to this assumption, rather than varying lithospheric strength. It appears that the initiation of subduction, and hence whether subduction zones form in an ‘internal’ ocean and introversion may occur, is no longer directly caused by the lithospheric strength but by when and where this weakening is introduced. Also, it appears that extroversion is inherently favored by the models because, starting from initial conditions, there are always already subduction zones in the external ocean, in order to disperse the continent, and it will be easier to maintain subduction zones rather than generate new ones.

Furthermore, oceanic lithosphere in the model does not newly form along spreading ridges, but rather it extends diffusely. Even though the authors assert that this model behavior does not affect the oceanic lithosphere ‘age’ distribution and hence results, it does not become clear that this is indeed the case. Rather, if there are spreading ridges in the middle of the oceans, the inferred age at the margins could on average be greater. This may influence where, in the model, subduction is initiated. In particular, if age at the margin of an internal ocean tends to become greater, it may increase the tendency for introversion rather than extroversion.

Overall, I find this an interesting paper, but find its prediction rather uncertain.

